# Endothelial dysfunction as a factor leading to arterial hypertension

**DOI:** 10.1007/s00467-022-05802-z

**Published:** 2022-11-21

**Authors:** Dorota Drożdż, Monika Drożdż, Małgorzata Wójcik

**Affiliations:** 1 Department of Pediatric Nephrology and Hypertension, Chair of Pediatrics, Pediatric Institute, Jagiellonian University Medical College, Krakow, Poland; 2 Deapartment of Pediatric and Adolescent Endocrinology, Chair of Pediatrics, Pediatric Institute, Jagiellonian University Medical College, Krakow, Poland

**Keywords:** Endothelial dysfunction, Hypertension, Cardiovascular risk, Children

## Abstract

Hypertension remains the main cause of cardiovascular complications leading to increased mortality. The discoveries of recent years underline the important role of endothelial dysfunction (ED) in initiating the development of arterial hypertension. The endothelium lines the interior of the entire vascular system in the body and acts as a physical barrier between blood and tissues. Substances and mediators produced by the endothelium exhibit antithrombotic and anti-inflammatory properties. Oxidative stress and inflammation are conditions that damage the endothelium and shift endothelial function from vasoprotective to vasoconstrictive, prothrombotic, and pro-apoptotic functions. A dysfunctional endothelium contributes to the development of hypertension and further cardiovascular complications. Reduced nitric oxide (NO) bioavailability plays an essential role in the pathophysiology of ED-associated hypertension. New technologies provide tools to identify pathological changes in the structure and function of the endothelium. Endothelial dysfunction (ED) contributes to the development of arterial hypertension and should be considered in therapeutic strategies for children with hypertension.

## Introduction

The blood vessels are built of many components, including endothelial cells, vascular smooth muscle cell, and adventitial tissues. Endothelium is the thinnest, but an especially important part of the vascular wall, built up of a single layer of cells. Until the 1980s, the role of the endothelium was understood as a semi-permeable thin barrier lining the vessels. Now it is considered the main and most important regulator of blood flow processes, and a particularly important element of homeostasis, with the ability to act in both sensory and effector capacities [1]. The discovery of the role of the endothelium, in particular its secretory function, allowed for a better understanding of the mechanisms leading to the development of the early stages of arterial hypertension [2, 3]. Endothelial cells perform unique functions that include fluid filtration, such as in the glomeruli of the kidneys, blood vessel tone maintenance, neutrophil recruitment, and hormone trafficking [4]. These functions are effected through the presence of membrane-bound receptors for numerous molecules including proteins, lipid-transporting particles, metabolites, and hormones, as well as through specific junctional proteins and receptors that govern cell–cell and cell–matrix interactions [5].

The main task of endothelial cells is to maintain and regulate blood flow to tissues to provide oxygen and nutrients. Under physiological conditions, endothelial cells regulate vasodilation. They also constitute a semi-permeable barrier between blood and peripheral tissues, exhibiting antithrombotic and anti-inflammatory properties. These properties result in vascular endothelium being called the gatekeeper of vessel health [6].

**Fig. 1:**
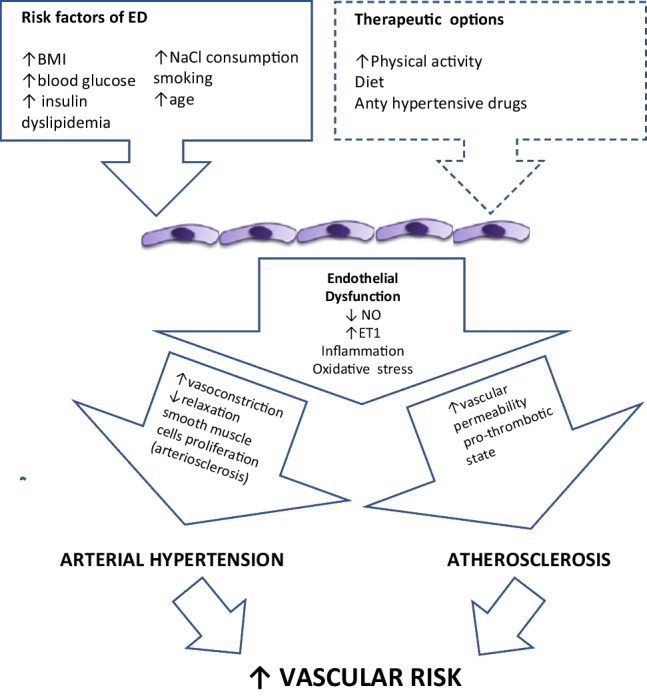
Key mechanisms leading to damage to the blood vessel wall

Diseases and processes that lead to the activation of inflammatory processes and oxidative stress adversely affect endothelial cells, causing dysfunction. Different cardiovascular risk factors, such as hyperglycemia, hyperlipidemia, hyperinsulinemia, insulin resistance, and hypertension, as well as other risk factors such as mental stress, aging, and exposure to anticancer drugs, participate in the onset of endothelial dysfunction (ED). Endothelial dysfunction is recognized as a hallmark of the vascular phenotype in patients with hypertension [7]. Moreover, recent studies have shown that ED contributes to both atherosclerosis and arteriosclerosis [8]. Also reduced nitric oxide (NO) bioavailability plays an essential role in the pathophysiology of ED-associated hypertension [9]. Additional factors are oxidative stress and vascular inflammation [10]. These may lead to changes in vascular resistance via two pathomechanisms: (1) abnormal vasoconstriction and relaxation and (2) promotion of vascular smooth muscle cell proliferation and activation of arteriosclerotic processes [10]. Activation of pro-inflammatory, pro-fibrotic, redox-sensitive, and growth/apoptotic pathways cause structural, functional, and mechanical changes with remodeling, calcification, and ED [11]. These biological events contribute to enhanced thrombosis, vasoconstriction, and inflammation. Animal research and the development of new techniques have allowed for a more detailed understanding of endothelial structure and regulatory mechanisms of various endothelial cell functions. Apart from arteriosclerosis, ED may also lead to increased permeability and formation of atherosclerotic plaques (Fig. 1).

Initially, research on ED was conducted mainly in adults. Only in recent years have results of research in children become available.

## Endothelial cell structure and physiology

Blood vessels are composed of three layers. The intimal monolayer of endothelial cells covers the entire vascular tree, from the heart to the smallest capillaries, forming the interior surface of all blood vessels [9]. Properly functioning endothelial cells play important roles in maintaining blood flow. Regulating blood pressure (BP) through vasodilation, they maintain a barrier between blood and adjacent tissues and have para- and autocrine functions. Endothelial cell phenotypes vary between different organs, between different segments of the vascular loop within the same organ, and between neighboring endothelial cells of the same organ and blood vessel type [12]. The endothelium of arteries and veins forms a continuous uninterrupted layer of cells, held together by tight junctions. The endothelium of capillaries may be continuous, fenestrated, or discontinuous, according to the needs of the underlying tissue. Fenestrated endothelium is characteristic of organs involved in filtration or secretion, including exocrine and endocrine glands, gastric and intestinal mucosa, choroid plexus, glomeruli, and a subpopulation of renal tubules [12]. The endothelial cells are covered with glycocalyx (GCX) (100–750-nm thick), which is a gel consisting of glycosaminoglycans (GAGs), and proteins, glycolipids, and glycoproteins. Heparan sulfate and hyaluronan are the main polysaccharides included in the structure. Heparan sulfate is the source of the GCX negative charge, while hyaluronan (a carbohydrate polymer) has the ability to bind to water and therefore is responsible for the gel-like characteristic [13]. The composition and dimensions of the GCX constantly change due to its replacement after damage from flowing blood and different thickness related to vessel dimension, varying from hundreds of nanometers to micrometers [14]. The GCX with bound proteins acts like a sieve preventing large molecules from passing through, and its degradation by activating inflammatory processes and increasing vascular permeability leads to important clinical consequences [13]. Activated endothelial cells and platelets release proheparanase which after transformation into active heparanase cuts heparan sulfate in the GCX and these fragments promote inflammation. Heparanase activity is also associated with the activation of the renin–angiotensin–aldosterone system. It has been shown in the animal model that antihypertensive drugs lisinopril and spironolactone are effective in reducing glomerular heparanase expression and restore the decreased heparan sulfate expression in the glomerular basement membrane [15]. The degradation of the GCX exposes adhesion molecules and the surface of endothelial cells and causes leukocyte–endothelial interactions [14]. Glycocalyx remodeling promotes interaction between endothelium and leucocytes enabling serum proteins, such as albumin and lipoproteins, to enter the subendothelial space. The discovery of the role of GCX damage in stimulating the inflammatory process and microalbuminuria explains the mechanisms of increased vascular permeability and the passage of substances from the blood into the sub-epithelial space. Reduced endothelial GCX thickness, which is responsible for increased transendothelial transport of LDL, as well as its intimal accumulation was detected in the carotid arteries in regions that are prone to atherosclerosis [13]. The relationship between hypercholesterolemia, endothelial damage, and the development of atherosclerosis was shown by the group of Chłopicki et al. in studies carried out in mice. They demonstrated in mice with hypercholesterolemia that GCX injury coincided with the impairment of endothelium-dependent vasodilation and increased endothelial permeability even before the development of atherosclerotic plaques [16]. Early changes were characterized by increased plasma concentration of biomarkers of GCX disruption (endocan), vascular permeability (angiopoietin 2), and endothelial inflammation (soluble vascular cell adhesion molecule 1). Hypercholesterolemia and increased stiffness of the endothelial cortex independently resulted in reduced release of NO and thus ED [17]. Glypican-1 is considered as a primary mechano-sensor for shear-induced NO production [18].

## Endothelial derived vasoactive mediators

The endothelium regulates vascular homeostasis through the release of a variety of autocrine and paracrine substances, such as NO, the main endothelium-derived vasodilatator, and endothelin-1 (ET-1), the main vasoconstrictor [19]. Although the biochemical factors involved in the regulatory mechanisms are numerous, NO and ET-1 are major players in this scenario [20].

## The leading role of NO as vasodilator

The increases in shear stress associated with blood flow and neurohumoral mediators through the activation of specific endothelial cell membrane receptors cause an instantaneous release of NO by the endothelial nitric oxide synthase (eNOS). Nitric oxide—a versatile signaling molecule—can be produced from L-arginine, S-nitrosothiols, and nitrate/nitrite in the vascular wall by different enzymes under various conditions. It regulates vascular tone by different signaling pathways (due to high diffusibility, it can freely transverse cellular membranes and regulate, e.g., soluble guanylyl cyclase (sGC)). It also decreases the intracellular concentration of calcium, causes relaxation of vascular smooth muscle, and thus is a potent vasodilator. Beyond its vasodilatory effects, NO has anti-atherogenic properties and inhibits platelet aggregation and adhesion, smooth muscle cell proliferation, leucocyte adhesion, vascular permeability, and inflammatory mechanisms [19]. Due to aging, obesity, and/or diseases (e.g., diabetes and hypertension), endothelial cells become dysfunctional, and as a result, the protective effect of NO is attenuated, which promotes vasospasm and emergence of atherosclerosis [21, 22]. The role of decreased NO level in developing hypertension was proven in the study with lenvatinib (an oral multi-kinase inhibitor)—an effective drug in the treatment of thyroid carcinomas. All of the 10 patients treated with lenvatinib exhibited significant hypertension. Serum nitrogen oxide concentrations were significantly decreased compared with pretreatment levels. In that study, authors provided the first demonstration that lenvatinib specifically causes hypertension via vascular endothelial dysfunction as a result of VEGF inhibition in human subjects [23]. Further endothelial-derived vasodilators include prostacyclin, which plays a synergistic role with NO, and bradykinin, which increases the release of NO. Reduced production and availability of NO are the main mechanism of the influence of ED on vasoconstriction, which, via increasing peripheral resistance, influences the development of arterial hypertension. The increasing stiffness of large arteries is believed to be the main cause of hypertension prevalence increase with age. In turn, in young people, one of the main mechanisms is the increase in peripheral resistance [24]. Other vasodilation mediators are the endothelial-derived hyperpolarizing (EDH) factors—molecules such as eicosatetraenoic acids, carbon monoxide, hydrogen sulfide, C-natriuretic peptide, and K ^+^ —which cause vasodilation by the activation of vascular smooth muscle cell K^ +^ channels, resulting in hyperpolarization and subsequently vasorelaxation [25]. The most recent findings underline that NO mainly mediates vasodilatation of relatively large, conduit vessels (e.g., epicardial coronary arteries), while EDH mediates factors in small resistance vessels (e.g., coronary microvessels). Small arteries and arterioles are the main source of resistance to blood flow, responsible for 70% of peripheral resistance. According to Poiseuille’s law, resistance is inversely associated with the fourth power of the blood vessel radius. As a result, minor decreases in the lumen will result in major increases in resistance to flow [24]. Endothelium-derived hydrogen peroxide (H_2_O_2_) is a physiological signaling molecule serving as one of the major EDH factors especially in microcirculations [26].

## Endothelin-1 and other vasoconstrictors

Endothelin-1 is one of the most potent endogenous vasoconstrictors, produced by endothelial cells, vascular smooth muscle cells, leukocytes, and macrophages. By activation of ETA and ETB receptors, it mediates the vasoconstrictor effect [6]. As demonstrated by Coelho and coauthors, the long-term induction of human ET-1 overexpression in mice caused sustained BP rise, small artery stiffening, endothelial dysfunction, and early kidney damage. These effects were reversed or reduced by a 2-week treatment with the ETA receptor antagonist atrasentan [27].

Hypoxia is one of the most important chemical stimuli of ET-1 release. An increase in ET-1 synthesis is also observed, however, in response to growth factors and cytokines, such as thrombin, tumor necrosis factor-α, interleukin-1, and insulin, but also to vasoactive substances such as norepinephrine, angiotensin II, vasopressin, and bradykinin [20]. The renin–angiotensin–aldosterone system also plays a significant role in vascular constriction. Almost all components of that system (with the exception of renin) are produced in blood vessels [6]. Angiotensin II (both produced locally and circulating) exerts its action through the AT1 and AT2 receptors promoting atherosclerosis, causing vasoconstriction and regulating expression of adhesion molecules (vascular cell adhesion molecule 1 (VCAM-1), intracellular adhesion molecule 1 (ICAM-1), P-selectin) and the secretion of cytokines, chemokines, and growth factors within the arterial wall [6]. Additionally, its action is linked to the vascular inflammation process. It promotes vascular infiltration by T cells and increases their interaction with the vascular endothelium [28].

## Main factors leading to endothelial dysfunction

### Oxidative stress

There is an important link between oxidative stress and endothelial dysfunction. Reactive oxygen species (ROS) significantly eliminate NO production [29]. Simultaneously, oxidative stress, inflammation, and ED together amplify the damaging process. Oxidative stress may be induced by prooxidant stimulants such as ROS, angiotensin II, ET-1, and inflammatory cells that dominate the antioxidant defense. Oxidative stress causes inflammation, which further leads to ED impairing vascular tone regulation as well as increasing susceptibility to formation of foam cells and adverse vascular remodeling [25]. Stimuli leading to vasorelaxation in the presence of intact vascular endothelium (such as acetylcholine) produce vasoconstriction when acting directly on the underlying smooth muscle cells in vascular areas with injured endothelium [30].

### Immune factors and ED

The earliest known relationship between the endothelium and the immune system was the adhesion of leukocytes and platelets and the rolling phenomenon [31]. Now it is clear that inflammation involves a coordinated interaction between the vessel wall, particularly the endothelium, and circulating immune cells. The process of rolling is followed by interaction of cell adhesion molecules (CAMs) including the intracellular adhesion molecules (ICAMs) 1–5 and VCAM-1 with leukocyte integrins including the lymphocyte function-associated antigen 1 and the very late antigen 4. Cell adhesion molecules are produced not only by endothelial cells, but also by pericytes and vascular smooth muscle cells. The interaction of selectins and CAMs with their ligand is often accompanied by diverse intracellular signaling events in both the vascular cell and the leukocyte [32]. Angiotensin II–induced hypertension is associated with an increase in vascular ICAM-1 expression, and this was attenuated by inhibiting the NADPH oxidase [33]. It was shown that mice lacking T and B cells (RAG-1^−/−^ mice) blunted hypertension and did not develop abnormalities of vascular function during angiotensin II infusion or desoxycorticosterone acetate (DOCA)–salt. Adoptive transfer of T, but not B, cells restored these abnormalities. On the other hand, hypertension increased T lymphocyte production of tumor necrosis factor α [28]. T lymphocytes contain a functional NADPH oxidase and an AT1 receptor, and angiotensin II stimulates T cell proliferation [34]. Oxidative stress may also contribute to immune system activation and further to the increase of vascular injury. As recently proposed by Wu et al., chronic vascular oxidative stress leads to the formation of immunogenic isoketal-protein adducts, which accumulate in dendritic cells and promote T cell activation. Activated T cells accumulate in perivascular tissues and release cytokines that enhance collagen deposition and aortic stiffening. This enhances pulse wave velocity. Kidney inflammation and fibrosis follows these events, leading to overt hypertension [35].

### Salt and ED

Recent studies have provided an explanation for the negative impact of excessive salt intake on the structure and function of endothelial cells. Under low salt conditions, the soft endothelial cortex (an actin-rich layer 50 to 150 nm beneath the plasma membrane) is easily deformable, the amount of shear stress–induced NO release is high. In the case of high salt-induced cortical stiffening, the NO release is reduced, leading to contracted smooth muscle cells and vasoconstriction [36]. In contrast, physiological elevations in plasma potassium concentration induce a plasma membrane electrical potential-dependent decrease in cortical stiffness and increased eNOS activity. High salt directly influences vascular endothelial cells leading to mechanical stiffening and a reduced NO release. This might lead to end-organ damage such as myocardial infarction, stroke, and chronic kidney disease [37]. Recent studies have shown that vascular endothelial cells express amiloride-sensitive Na^ +^ channel activity (EnNaC). Under low salt conditions, the endothelial cell is protected by a well-developed GCX with an optimum of negatively charged proteoglycans and a low number of EnNaC in the plasma membrane. Thus, plasma Na ^+^ is buffered and the access of Na ^+^ to the respective channels of the endothelium is limited, resulting in a soft cortex. In the case of high salt concentrations, a poorly developed GCX with a reduced number of negatively charged proteoglycans can be found, while the number of EnNaC is increased. Thus, plasma Na ^+^ has facilitated access to the sodium channels of the endothelium and cortical stiffening occurs. Furthermore, the genotype of endothelium influences these processes, e.g. local salt-induced ENaC (epithelial sodium channel) activation may result in considerable systemic effects. In the short term, this mechanism might be helpful for the stabilization of BP due to the loss of extracellular volume and an increase of plasma Na ^+^ . However, in the long term, these pathophysiological processes may be harmful. Therefore, an early identification of gene-associated salt sensitivity and treatment with direct (amiloride) and indirect (spironolactone) ENaC inhibitors might be of great importance in the treatment and prevention of cardiovascular diseases [36]. In young normotensives, salt loading not only impaired endothelial function but also left ventricular relaxation and electric repolarization [38]. Interestingly, there is a link between the immune system and high salt diet. It has been shown in a mouse model that deficiency of IL-17A or IL-6 leads to a minimal increase in BP in response to a high salt diet and angiotensin II [39].

## Endothelium dysfunction as a cardiovascular risk factor

The main goals in hypertension treatment are normalizing BP and decreasing cardiovascular risk. The detrimental effect of traditional cardiovascular risk factors such as age, gender, body mass index (BMI), waist circumference, systolic BP, fasting glucose, total cholesterol, and smoking on impairment of endothelial function measured by flow-mediated dilatation (FMD) was proven in adults [40]. Clinical trials have also shown an association between markers of ED and increased cardiovascular risk. Albuminuria is not only a sign of kidney damage in hypertension but also a symptom of systemic ED. In their pooled analysis of over two million participants from the general population, high risk, and chronic kidney disease cohorts, Nitsch and coauthors found increased risk of all-cause and cardiovascular mortality and kidney failure with lower estimated glomerular filtration rate and higher albuminuria in both sexes [41]. Compared with a urinary albumin:creatinine ratio of 5, the adjusted hazard ratio for all-cause mortality at urinary albumin:creatinine ratio 30 was 1.69 (1.54 to 1.84) in women and 1.43 (1.31 to 1.57) in men. In diabetic patients with albuminuria, ED of peripheral arteries independently predicted future cardiovascular events (e.g., death, acute coronary events, stroke) [42].

The brain is an early target for organ damage by elevated BP. In adults, hypertension is the main risk factor for stroke, whereas in hypertensive children, it is neurocognitive dysfunction [43]. Hypertension causes vascular brain injury directly (small vessel disease) or by promoting atherosclerosis or cardiac damage. Preliminary studies in children on cerebrovascular reactivity showed decreased vasodilatory and vasoconstrictor ability of the cerebral arterioles. Cognitive deficits in hypertensive individuals might be the result of decreased ability to enhance cerebral blood flow in response to increased neuronal activity [43]. As shown by Georgakis et al., albuminuria was independently associated with cerebral small vessel disease, which was diagnosed using magnetic resonance imaging [44]. The authors suggested that brain microvasculature damage could be evaluated via peripheral systemic microvascular disease biomarkers and that cerebral small vessel disease mediates the previously described association between albuminuria and dementia.

The importance of uremic toxins in endothelial damage and the role of ED in increased cardiovascular risk in patients in advanced stages of chronic kidney disease are also emphasized [45].

## Methods of endothelium assessment

### Biomarkers

High concentrations of circulating markers (adhesion molecules, von Willebrand factor (vWF), syndecan-1, soluble thrombomodulin (TM)) are found in ED [46, 47]. Endothelial microparticles (EMPs), small-membrane vesicles that are shed from the surface of endothelial cells, correlated significantly with systolic and diastolic BP in hypertensive patients and also with other parameters of EDC activation (sVCAM-1, sICAM-1, vWF) [48]. Endocan levels were significantly higher in patients with hypertension than in controls [49, 50]. According to Leite and coauthors, endocan fulfills the criteria for an ideal endothelial biomarker, because of high reproducibility, readiness, feasibility, and low costs of measuring endocan by ELISA kits [51]. One of the molecules shed from the injured endothelium is TM. In children with chronic kidney disease, TM levels strongly depend on kidney function parameters, oxLDL levels, and 24-h systolic and mean BP values. In children on chronic dialysis, angiopoietin-2 was identified as a marker of ED and cardiovascular disease [52].

The results of clinical trials on biomarkers of ED in children are presented in Table 1.

**Table 1:** The results of clinical trials on biomarkers of endothelial dysfunction in children

Endothelial mediated processes	Biomarkers	Clinical studies in children
Vasodilation	Nitric oxide (NO) Prostacyclin Endothelial-derived hyperpolarizing factors (EDHF) Adenosine	Serum NO levels were higher in prehypertensive children than in hypertensive children [53] Reduced NO production in monocytes in children with OSA and ED [54] Genetic variants in the *NOS1* and *EDN1* genes appear to account for important components of the variance in endothelial function [55]
Vasoconstriction	Endothelin-1 (ET-1) Angiotensin II Thromboxane A2 Reactive oxygen species	Plasma ET-1 concentrations in prehypertensive and hypertensive adolescents were higher than in controls [53]
Glycocalyx disruption	Syndecan Endocan	In adolescents with excess weight positive correlations between syndecan-1 and serum creatinine and triglycerides were found [56] The endocan levels were significantly elevated in the pediatric kidney transplant patients who had hypertension and a loss of kidney function [57] Endocan was increased almost threefold in children with metabolic syndrome compared to healthy controls [58] In children undergoing cardiac surgery on cardiopulmonary bypass, increases of circulating hyaluronan and syndecan-1 were present [59] Children with Kawasaki disease, especially with coronary involvement, had higher levels of syndecan-1 in plasma compared healthy controls [60] In children with type 1 diabetes an inverse correlation between serum glucose levels and glycocalyx thickness was observed [61]
Endothelial inflammation	Soluble vascular cell adhesion molecule 1 (sVCAM-1), the soluble form of E-selectin (sE-sel), Soluble intercellular adhesion molecule 1 (sICAM-1) Angiopoietin-2 (Ang-2)	In children with type 1 diabetes mellitus a significant positive correlation between sE-selectin and systolic as well as diastolic pressure loads during the day period in ABPM was found [62] The Ang-2/Ang-1 ratio was higher in children with septic shock. Ang-2/Ang-1 was associated with higher vasoactive agents, longer ICU length of stay, and correlated with the severity of illness score [63] Ang-2 is a marker for cardiovascular disease in children on chronic dialysis and may act as an anti-angiogenic and pro-inflammatory effector [64]
Endothelial permeability	Angiopoietin 2 (Ang-2) The soluble form of FMS-like tyrosine kinase (sFLT-1)	sFLT-1 may contribute to pathogenesis of albuminuria in sickle cell disease patients [65]
Angiogenesis	The soluble form of FMS-like tyrosine kinase (sFLT-1)	sFLT-1 may contribute to pathogenesis of albuminuria in sickle cell disease patients [65]
Hemostasis	von Willebrand factor (vWF), Tissue plasminogen activator (t-PA), Plasminogen activator inhibitor 1 (PAI-1)	Obese children had higher risk of developing early platelet activation-associated factors: leptin, vWF, UA, and HDL [66] PAI-1 levels were significantly elevated in children with type 1 diabetes, especially with micro-vascular complications, compared to controls [67]
Messaging, cell activation, apoptosis, tissue degeneration	Cell membrane microvesicles	Higher concentrations of endocan, angiopoietin-2, annexin-V-positive microvesicles and endothelial microvesicles expressing MadCAM1 and lower VEGF was present in the plasma of critically ill newborns with multiple organ dysfunction on ECMO [68] Endothelial microvesicles were found in neonates and children with vasculitis and blood group incompatibility [69, 70

*OSA* obstructive sleep apnea; *ED* endothelial dysfunction; *NOS* nitric oxide synthase; *EDN1* endothelin-1 gene; *ECMO* extracorporeal membrane oxygenation; *UA* uric acid; *HDL* high-density lipoprotein

### Invasive and non-invasive techniques of endothelial function evaluation

To measure changes in vessel size, invasive methods require intracoronary or intrabrachial infusions of vasoactive agents followed by quantitative coronary angiography or intra-vascular ultrasound (IVUS) [10]. Endothelial function can be assessed in humans by FMD, which uses non-invasive ultrasound to measure the percentage of dilatation of the brachial artery in response to blood flow and is due to an increased endothelial formation of NO in response to shear stress. The diameter of the brachial artery is measured before and after a forearm BP cuff occlusion-induced reactive hyperemia. The BP cuff is inflated to suprasystolic pressures for 5 min. The degree of artery dilatation after hyperemia (expressed as % of pre-hyperemia diameter) reflects arterial endothelial NO release. A standard method of endothelial function evaluation, FMD of the brachial artery requires experience and is operator dependent.

One non-invasive method is reactive hyperemic peripheral arterial tonometry (RH-PAT). As shown by Mueller et al., reactive hyperemic index (RHI) is age- and sex-dependent in school children aged 10–17 [64]. Reduced vasodilatation is an initial stage in the development of hypertension and has been demonstrated in clinical trials in various risk groups for hypertension in children and adolescents. FMD was lower in non-obese (8.5 ± 4.5%) and obese (8.1 ± 4.9%) newly diagnosed hypertensive adolescents than in a healthy control group of adolescents (12.5 ± 4.9%) [71]. Jurko and coauthors found impaired FMD in children with hypertension as well as in children with white-coat hypertension [72]. In children with chronic kidney disease, which is a well-known cardiovascular risk factor, FMD was reduced (10.6 ± 4.9% vs. 18.9 ± 4.1%) [73]. In obese children, FMD was markedly reduced, however, positively correlated with HDL [74]. On the other hand, studies in adults indicate a lower effect of ED in advanced stages of NT and vascular remodeling. In the FMD J (flow-mediated dilatation Japan) study, systolic FMD in untreated adult subjects with hypertension was associated with BP value, contrary to treated hypertensive patients, where no such significant relationship was found [75]. The authors underlined that endothelial function assessed by FMD in hypertensive patients was impaired regardless of BP control achieved with antihypertensive drugs. In a prospective study in adults with essential hypertension and no- or 1-target organ damage (TOD) at baseline, impaired FMD led to thicker intima-media thickness, faster pulse wave velocity, and increased urinary albumin:creatinine ratio at the end of the follow-up period, even after adjusting for age and sex [40]. It indicates the role of ED in the early phase of vascular injury. Matsuzawa et al. performed a systematic review and meta-analysis to investigate the prognostic magnitude of noninvasive peripheral endothelial function tests, brachial artery FMD, and RH-PAT. The meta-analysis found that both brachial FMD and digital RH-PAT have significant predictive value for future cardiovascular events. Greater vasodilation capacity (1 SD increase in FMD) was associated with 50% lower risk of cardiovascular events [76].

## Treatment

### Non-pharmacological treatment of arterial hypertension and endothelium

Numerous studies have shown a beneficial effect of nutritional factors, e.g., through antioxidant activity on the endothelium. Furthermore, physical exercise and healthy nutrition are the pillars of non-pharmacological cardiovascular therapy. Antihypertensive drugs and lifestyle modifications can repair and ameliorate vascular damage. These vascular actions, together with BP-lowering effects, reduce cardiovascular risk and complications of hypertension.

### The role of diet

In the position document of the European Society of Hypertension, the use of nutraceuticals (beetroot juice, tea, and coffee as antioxidant-rich beverages, magnesium, potassium and vitamin C, resveratrol present in red wine and more specifically in red grape skin) is suggested in patients with high–normal BP [77]. Sebastian and coauthors hypothesized that Americans on average consume suboptimal amounts of potassium and BP-lowering phytochemicals, and physiologically excessive amounts of sodium, and that such a diet leads to essential hypertension through oxidative stress-induced vascular endothelial and smooth muscle dysfunction [78]. The use of healthy diets, rich in vegetables and fruit, has a proven role in reducing cardiovascular risk in adults. What is more, their beneficial effects on the endothelium have also been demonstrated. The recommended diets are the Mediterranean Diet (MedDiet); DASH, dietary approach to stop hypertension; the portfolio diet; the vegetarian diet; the Nordic diet; and low-carbohydrate diets [79]. As shown by meta-analysis, portfolio diet in patients with diabetes type II resulted in significant decreases in LDL level and increases in FMD [80]. In turn, the MedDiet intervention improved FMD regardless of healthy status, BMI, or age of adult participants [81].

Weight reduction in obese children resulted in improved endothelial function as measured by the EndoPAT device. This improvement was less pronounced in children who had persistent sleep apnea despite losing weight [82]. In a clinical study after a 4-week run-in period during which fruit and vegetable intake was limited to 1 portion per day, participants were randomized to consume either 1, 3, or 6 portions daily for the next 8 weeks. For each 1-portion increase in reported fruit and vegetable consumption, there was a 6.2% improvement in forearm blood flow responses to intra-arterial administration of the endothelium-dependent vasodilator acetylcholine [83]. This may be related to high polyphenol content in fruit and vegetables, which can increase NO bioavailability. It was shown that polyphenols, such as resveratrol, exhibit vasculoprotective properties as they decrease cortical stiffness and induce NO release. The largest group among polyphenols are flavonoids, such as quercetin and kaempferol, which are found in most kinds of plants. Various groups of polyphenols are found in, for example, tea (theaflavins), colorful fruit (anthocyanidins) or wine (resveratrol). In healthy subjects, polyphenol-rich products have been shown to increase FMD by relatively low doses, such as the consumption of two glasses of red wine with or without alcohol, or of a flavonoid-rich dark chocolate for 2 weeks. A meta-analysis performed by Ebaditabar et al. showed that acute and chronic consumption of dark chocolate and flavonoids have beneficial effect on FMD [84]. In animal hypertension models, biological actions of different flavonols (quercetin—the greatest vascular activity; fisetin, kaempferol) such as vasorelaxation, antioxidant activity by scavenging superoxide and activating antioxidant defense system and anti-inflammatory activity, were shown [85].

Flavonols have vasoprotective properties and are a potential group for use in treatment. Alpha-lipoic acid (ALA) is a commonly used dietary supplement exerting antioxidant and anti-inflammatory effects. In a group of obese children after 3 months of ALA supplementation, the basal and peak diameter of brachial artery significantly increased [86]. Intake of polyphenol-rich products like cocoa, tea, grapes, berries, and nuts positively affects endothelial cells and results in increased production of NO.

As has been shown in experimental and clinical studies, polyphenols exert their beneficial effects through reducing ED, platelet activation, vascular oxidative stress, overactivation of the local angiotensin system, and increased pro-thrombotic responses associated with cardiovascular risk factors [87].

### The role of physical activity

Multiple studies in adults have shown that exercise training is associated with modest but significant reductions in both all-cause and cardiovascular mortality, increased exercise capacity, and improved quality of life [88]. Effects of exercise on the vascular endothelium are mediated by intermittent increases of laminar shear stress which activates nitric oxide synthase and leads to higher NO production. Paula et al. provided, in an experimental rat model, a molecular basis for exercise-induced NO bioavailability and higher antioxidant defense in femoral arteries [89]. Exercise training reduces resting BP, heart rate, and reverses cardiac hypertrophy, improves the response of the microvasculature to insulin, and increases the microvascular network via angiogenesis and arteriogenesis [90]. Physical exercise activates metabolic processes in various tissues and organs, which, through the production of signaling molecules, hormones, and cytokines named “exerkines,” exert beneficial systemic effects, e.g., increase insulin sensitivity or lead to inter-organ crosstalk. Exercise can be effective in reducing BP, as shown by the acute post-exercise hypotension which may last for up to 24 h. Multiple studies including epidemiological studies, controlled clinical trials, and meta-analyses showed that regular aerobic exercise results in a BP lowering effect of 3 to 7 mmHg, reaching up to 15 mmHg [91]. What is interesting is that these results showed endothelium-dependent vasodilation evaluated using brachial FMD is maintained or improved following acute aerobic exercise in moderately trained participants, but not in well-trained participants, especially if they are engaged in resistance training [92]. In adults, different exercise training modalities (aerobic, resistant, and combined) performed for 40 min twice a week for 8 weeks were similarly effective in improving endothelial function measured by FMD [93]. High school students randomized to 60-min physical exercise (PE) at school daily (intervention group) had higher RHI (reactive hyperemic index) than the control group—2 units of 45-min PE weekly [71]. In recently published guidelines, the World Health Organization (WHO) recommends at least 150 to 300 min of moderate aerobic activity per week (or the equivalent vigorous activity) for all adults, and an average of 60 min of moderate aerobic physical activity per day for children and adolescents [94].

## Pharmacological treatment of arterial hypertension and endothelial dysfunction

Antihypertensive agents with the capacity to reverse ED as well as reducing BP (Table 2) may reverse or prevent the progression of atherosclerosis and thereby reduce the risk of serious complications of hypertension, such as myocardial infarction and stroke [11]. Angiotensin-converting enzyme inhibitors (ACEIs), angiotensin II receptor blockers (ARBs), and calcium channel blockers have all been shown to improve endothelial function with associated improvements in markers of oxidative stress. Drugs used in nephroprotection—ACEIs and ARBs—in addition to their hypotensive effect, have a documented beneficial effect on the GCX by inhibiting heparinase, an enzyme that cleaves heparan sulfate side chains. A decreased expression of heparan sulfate in the glomerular basement membrane is commonly present in glomerular diseases. Beta-blockers, despite lowering BP, generally do not improve endothelial function. Nebivolol and carvedilol are the exceptions: nebivolol has NO donor properties and carvedilol may act as a scavenger of oxygen free radicals [11].

**Table 2:** Antihypertensive drugs and beneficial vascular effects according to [4]

Drug class	Possible beneficial vascular effects
ACE inhibitors	Vasodilation Increased nitric oxide Anti-inflammatory Reduced reactive oxygen species
Angiotensin II receptor blockers	Vasodilation Increased nitric oxide Anti-inflammatory Reduced reactive oxygen species
Calcium channel blockers	Improved cellular redox state
Diuretics	Reduced arterial stiffness Anti-inflammatory/fibrotic (via natriuresis)
Mineralocorticoid receptor antagonists	Anti-inflammatory Anti-fibrotic
β-Blockers (nebivolol, carvedilol)	Increased nitric oxide Reactive oxygen species scavenger

*ACE* angiotensin-converting-enzyme

## Conclusions

Endothelial dysfunction is associated with almost all cardiovascular risk factors, precedes the development of atherosclerosis, predicts cardiovascular events independently of classical risk scores, might identify a subset of patients in which conventional treatment is not sufficient and accompanies prehypertension.

While many systems contribute to BP elevation, the vascular system is particularly important because vascular dysfunction is a cause and consequence of hypertension. Pharmacological substances which are able to improve endothelial structure and function could achieve a new importance in the prevention and treatment of vascular diseases. Antihypertensive drugs that influence vascular changes associated with high BP have greater efficacy for reducing cardiovascular risk than drugs that reduce BP, but have little or no effect on the adverse vascular phenotype. Healthy diet with decreased salt and increased fruit and vegetable consumption, as well as physical exercise, improve endothelial function and reduce BP and should therefore be strongly encouraged in both the wider community and patients with hypertension.

## Key summary points

Endothelial dysfunction:

- Is associated with almost all cardiovascular risk factors.
- Exhibits vasoconstrictive, pro-thrombotic, and pro-apoptotic properties.
- Reduced nitric oxide (NO) bioavailability plays an essential role in the pathophysiology of ED-associated hypertension.
- Precedes the development of atherosclerosis.
- Endothelial guided therapy (non-pharmacological and pharmacological) is of greatest importance in children and in the early stages of hypertension.

## Multiple choice questions (answers are given after the references)

- 1. Indicate incorrect answer for nitric oxide (NO):
  - A) NO is the main endothelium-derived vasodilatator
  - B) Potassium has an opposite function to NO
  - C) The main substrate to produce NO is arginine
  - D) NO inhibits platelet aggregation and adhesion, smooth muscle cell proliferation. and leucocyte adhesion
  - E) NO inhibits vascular permeability and inflammatory mechanisms
- 2. Which antihypertensive drug has no beneficial effect on endothelial cells:
  - A) Valsartan
  - B) Amlodipine
  - C) Ramipril
  - D) Metoprolol
  - E) Nebivolol
- 3. What is the biomarker of glycocalyx damage?
  - A) Syndecan-1
  - B) Angiopoietin-2
  - C) Angiotensin II
  - D) A + B
  - E) A + C
- 4. Which of the following statements about glycocalyx (GCX) is true:
  - A) It has only a mechanical function
  - B) Injury to the GCX increases the risk of atherosclerosis
  - C) Decreased heparanase activity leads to GCX damage
  - D) Loss of GCX decreases transcapillary albumin transport
  - E) GCX damage does not affect vascular permeability
- 5. Which of the following methods is not used to evaluate endothelial function in children in studies?
  - A) FMD
  - B) Albuminuria
  - C) Endocan level
  - D) Thrombomodulin level
  - E) Quantitative coronary angiography

## Data Availability

Not applicable.
